# Development and Validation of a Language Screening for Implementation in Pre-School Settings

**DOI:** 10.3389/fpubh.2022.866598

**Published:** 2022-06-22

**Authors:** Daniel Holzinger, Christoph Weber, Bettina Diendorfer

**Affiliations:** ^1^Institute of Neurology of Senses and Language, Hospital of St. John of God, Linz, Austria; ^2^Research Institute of Developmental Medicine, Johannes Kepler University Linz, Linz, Austria; ^3^Institute of Linguistics, University of Graz, Graz, Austria; ^4^Department for Inclusive Education, University of Education Upper Austria, Linz, Austria

**Keywords:** language screening, language disorder, LOGiK-S, validity, feasibility, pre-school

## Abstract

**Background:**

To prevent or mitigate long-lasting learning problems and emotional, behavioral, and social-adaption difficulties associated with language disorders, age-appropriate German language competence at school entry level is essential. Therefore, universal screening of children in their penultimate year of pre-school has been established in Upper Austria. So far, the screenings administered by speech and language pathologists to identify risk of language disorder (LD) were not based on standardized materials.

**Objective:**

To develop a screening instrument to identify increased risk of LD and to evaluate its validity and feasibility within the constraints of regular universal pre-school language screening.

**Design:**

A two-component screening instrument including direct assessment of expressive and receptive grammar was used in a sample of 374 children with German as their dominant language attending a public pre-school in their penultimate year (age 4-5 ½ years) in the state of Upper Austria. Assessment by use of standardized German language tests including a variety of linguistic domains was considered reference standard for diagnosing LD. Feasibility was assessed by a self-developed questionnaire completed by the administrators of the screening.

**Results:**

The combination of the expressive and receptive grammar scales demonstrated excellent accuracy (area under the curve score 0.928). A cut-off of 18 resulted in a failing rate of 21.8% and showed good sensitivity (84.2%) and specificity (85.3%). Acceptance by children and testers, time-economy and sustainability of the screening were mostly rated as high.

## Introduction

The international CATALISE consortium (1) recently addressed the issue of terminology and definition of problems with language development, by defining diagnostic criteria for the newly termed Developmental Language Disorder (DLD) by the CATALISE Consensus. The new term DLD refers to a language disorder (LD) that emerges during development and is not associated with known biomedical conditions. DLD is a heterogeneous condition, which can affect language production and/or comprehension and different linguistic domains (lexical, morpho-syntactic, pragmatic). The new definition of DLD does not preclude the co-occurrence with other neurodevelopmental conditions, the presence of environmental risk factors or require a mismatch between verbal and non-verbal cognition. In addition, the consensus statement agreed on the serious nature of language problems with a significant impact on everyday social interactions or educational progress and poor prognosis of LD. What the consensus statement did not define is the extent of language difficulties in mode (receptive and/or expressive) and linguistic dimension (phonology, vocabulary, morphology, syntax, pragmatic s). Therefore, DLD remains a clinical diagnosis, where professionals need to be able to recognize language deficits associated with functional impairment and the potential of these conditions to become chronic with an increased risk of learning and mental health problems.

With language abilities at least 1.5 SD under those of peers in at least two of the five linguistic domains, Norbury et al. (2) found a prevalence of LD of any origin of about 10% (7.58% specific with unknown origin and 2.34% non-specific with medical diagnosis), which makes LD one of the most common developmental problems in childhood. Similarly, earlier studies that assume language abilities around 1.25 SD below the norm in two linguistic domains, expect a prevalence rate of 5-8% of specific LD in children speaking English (3, 4), English or French (5) or German (6).

Children with LD are at high risk of difficulties in academic and vocational qualification (7, 8), mental health problems and social adaptation difficulties (9–11). Early identification of LD may help children to access specialized educational (9), therapeutic (12) and parent-implemented (13) intervention to support them to improve their language skills by school entry and to reduce the risk of neuropsychological sequelae. As a consequence, a system for a universal language check-up has been established in the State of Upper Austria since the mid 90's administered by speech and language pathologists. In Upper Austria, a federal state with a population of 1.45 million inhabitants, all children (about 14.000/year at the time of data collection) are assessed in their penultimate year of pre-school for speech and language development every year. Up to this point, speech and language pathologists are faced with the challenge of accurately identifying the children with the highest risk of persisting language difficulties and need of language intervention. The challenges concern the lack of a generally accepted definition of what constitutes a LD and the lack of a standardized and feasible procedure for language screening. Another challenge concerns the high variability of language development during the early years with a high proportion of children with initially poor language catching up before school entry (14–16) and others manifesting deterioration in the trajectory of language development over time. Whereas some studies have demonstrated relatively stable trajectories of language development from the age of 5-6 years (2, 17), more recent population cohort studies have shown that the degree of variability in child language pathways even after the age of 4 or 5 years might have been underestimated suggesting the necessity of continuous surveillance of language development and environmental risk factors (18, 19).

In 2006, Nelson et al. concluded their review for the US Preventive Services Task Force advising against universal language screenings because of many methodological problems they had identified in language intervention and outcome studies (20) provided an update to the (21) systematic review reporting sufficient accuracy of some screening tools for the identification of children with LD but highlighting a lack of studies demonstrating their feasibility in primary care settings. They also reported that some treatments for children 5 years and younger might be effective but criticized the lack of well-conducted studies. As consequence, the US Preventive Service Task Force continued not to recommend universal language screenings for language delay (22). For the German speaking community, the German Institute for Quality and Efficiency in Health Care (IQWIG; Institut für Qualität und Wirtschaftlichkeit im Gesundheitswesen) also criticized the lack of evidence for long-term outcomes of language therapy. Following international systematic reviews (21, 23) the implementation of universal language screenings in Germany was not recommended (24).

So far, no standardized language screening instrument validated for use in Austrian pre-schools has been available. In Germany, several federal states commissioned research institutes to generate standardized language measures for the identification of language delayed children [i.e., Sismik & Seldak in Bavaria from (25, 26); HASE in Baden-Wuerttemberg from (27); KiSS in Hessia from (28) or Delfin 4 in North Rhine-Westfalia from (29) to name some]. Nevertheless, an analysis of the German Mercator- Institute for language promotion and German as second language ascertained insufficient quality and efficacy for all the screenings, mainly because of lack of sufficient validity and objectivity and the exclusion of multilingualism (30).

In Upper Austria, the request for a standardized procedure to be used within the regular universal check-ups in pre-schools, led to the LOGiK-S (Logopädie im Kindergarten—Screening) project. The new measure assesses language skills in Standard Austrian German, the variety of Standard German spoken in Austria in more formal situations (eg in schools and in the media) and with the highest sociolinguistic prestige. In less formal situations most Austrians use dialectal variations of German (Bavarian and Alemannic). The minor differences between Austrian German and Standard German spoken in Germany relate particularly to vocabulary and idiomatic expressions and less to language structure.

Our aim was to develop an accurate screening tool for the identification of high risk of LD (of unknown origin or associated with other biomedical conditions) in Austrian children and to evaluate its feasibility in the pre-school community setting.

## Methods and Procedures

### Participant Recruitment

In summer 2012 and summer 2013, the public pre-schools in the city of Linz and in the whole state of Upper Austria were invited to participate in the project LOGiK-S (logopedics in kindergarten–screening) with the aim to develop a standardized instrument for language screening. In total, 31 pre-schools (14 of them well spread over different districts of Linz and 17 in the districts of Upper Austria) agreed to participate in the study. The recruitment of pre-schools in two consecutive years was due to limited human resources in the research team and to avoid overburdening the collaborating pre-schools. The managers of the pre-schools disseminated information about the project to all parents of children in their penultimate year of pre-school (age of 4-5 ½ years; Children attending their penultimate year of pre-school in the school year 2012/2013 are hereafter labeled as Cohort A and children attending their penultimate year of pre-school in the school year 2013/2014 are labeled as Cohort B) and asked for written consent for their children's participation. Overall, 423 monolingual children with German as their only language (as reported by the pre-school teachers) were eligible to participate. 97.9% of the parents (total *n* = 414, *n* = 208 in Cohort A and *n* = 206 in Cohort B) gave their written permission for inclusion in the research study. Testing was conducted in the first half of the school year (October 2012 to April 2013 for Cohort A and September 2013–March 2014 for Cohort B). We excluded children with incomplete data on the screening and reference tests (*n* = 13 in Cohort A and *n* = 16 in Cohort B), children with a time interval between screening and reference test of more than 60 days (*n* = 7 in Cohort B) and children outside the target age range (*n* = 1 in Cohort A and *n* = 3 in Cohort B). The remaining *n* = 374 children (*n* = 194 in Cohort A and *n* = 180 in Cohort B) were included in this study. Table 1 provides an overview of the sample characteristics. Half of the children were girls (50.0%). The mean age was 55.66 months (SD = 4.01), whereas Cohort B was about 1 month older than Cohort A (t = 2.100, *p* < 0.05). Compared to the Upper Austrian parent population (29), the share of parents with university degree was overrepresented in the sample [36.1% vs. 25%; χ^2^(3) = 28.725, *p* < 0.001], which can be probably be explained in part by the exclusion of children with first languages other than German, whose parents are less likely to have a university degree [(29) Population data on parental education are not available for German-speaking children]. Moreover, there were some differences in parental education between Cohort A and Cohort B (see Table 1), most likely due to different catchment areas of pre-schools. However, these differences were not significant [χ^2^(3) = 7.604, *p* > 0.05]. For the analyses of this paper, we used pooled data (i.e. we analyzed cohort A and cohort B together) to maximize statistical power. Data pooling would also increase external validity, as the pooled sample is likely to be more heterogeneous in terms of individual characteristics than the single cohorts.

**Table 1:** Participant characteristics.

	**Cohort A (2012)** ***n* = 194**	**Cohort B (2013)** ***n* = 180**	**Total** ***n* = 274**
Number of pre-schools	17	18	31
Females %	50.5%	49.9%	50.0%
Age M (SD)	55.24 (4.02)	56.11 (3.94)	55.66 (4.01)
*Highest parental education^*a*^ %*			
Compulsory education (or below)	6.4%	2.5%	4.6%
Vocational education	33.5%	39.9%	36.4%
University entrance level	19.7%	26.%	22.8%
University degree	40.4%	31.0%	36.1%

*^a^he highest education of the two parents was used*.

The study project (cohorts A and B) was approved by the hospital's ethic commission “Ethikkommission Barmherzige Schwestern und Barmherzige Brüder”.

### Measures

#### Construction of the Screening Measures

At the age range relevant for the current study (4 ½ to 5 years) the primary markers of LD in German are deficits in morphosyntax, such as lacking or incorrect inflection of verbs (31), subject-verb-agreement (30) or use of function words (31). In addition, clinical experience shows that the valid assessment of grammatical skills is less time-consuming than the assessment of vocabulary. An expressive and receptive screening scale was developed because LD can affect the production and comprehension of language structures. In addition, assessments of language reception do not require the child's active production of language and therefore, higher acceptance of the receptive language assessment was anticipated. For both screening scales, grammatical structures that are usually acquired at pre-school age were selected. Based on the available literature on acquisition of German grammar (32–37), morphosyntactic structures with different degrees of complexity were selected. Children in their penultimate year of pre-school were chosen as the target group by request of the public authorities, following the tradition of universal language screening before the final year of pre-school, when—if necessary—intervention can be implemented before school entry.

#### Expressive Grammar Screening

The expressive grammar (EG) scale includes sentence completion tasks eliciting spoken phrases from the child with the help of predetermined sentence patterns. The scale includes 17 items. The tester successively presents two pictures, separated by a dividing line. The grammatical pattern structure is introduced with reference to the first picture (e.g., “Look! This is Tobias. He drinks juice.”). After that, the child completes the sentence presented along with the second picture eliciting the same grammatical target structure (e.g. “*And this is Maria. She* …”–target structure: verb second position). Child utterances are scored as correct, when the child is able to produce the target grammatical structure. Errors beyond the targeted grammatical structure are negligible. To facilitate the scoring (0/1 points) of the expressive language items, a collection of correct and incorrect answers is provided. Notably, in cohort A, the screening scale comprised a total of 27 items. The final set of 17 items for measuring EG was selected based on the item statistics (difficulties, item-scale correlation) and the feedback of speech therapists who administered the screenings.

#### Receptive Grammar Screening

The receptive grammar (RG) scale includes 14 items, again ranked by anticipated increase in complexity, following German language acquisition research. Single sentences are read aloud by the administrator of the test and the child is asked to point to the corresponding picture from a selection of four with well-chosen semantic and grammatical distractors. The test items assess comprehension of different syntactic (e.g. “*The boy slides and the girl swings*”—coordination) or morphological structures (e.g. “*He gives her the book*.”—pronouns). Similar to the EG scale development, an initial number of 20 items was reduced to 14 items based on results of cohort A.

#### Reference Language Tests

Without an accurately defined gold standard for LD in the literature, LD was operationalized by significant deficits (-1.25 standard deviations below the norm) in at least two of the three linguistic dimensions of EG, RG and expressive vocabulary (compare 2–4).

(1) Expressive grammatical skills were assessed by the plural and case (accusative and dative) marking subtests of the PDSS [(38); *Patholinguistische Diagnostik bei Sprachentwicklungsstörungen*] as well as the subtests for comparatives, superlatives and participle perfect formation of the ETS 4-8 [(39); *Entwicklungstest Sprache für Kinder von 4 bis 8 Jahren*]. Following the results of a principal component analysis (PCA; one component with an eigenvalue of 2.27, 57% explained variance; loading between .71 and .83), we saved the component score (z-score with M = 0 and SD = 1) to be used as a single EG measure. Internal consistency (Cronbach's α) was good at .73. Children were classified as atypical in EG, if they scored in the bottom 10% (-1.25 SD) of the component score.

(2) The TROG-D [German version of the Test the Reception of Grammar; (40)] assesses the understanding of German grammar. Although the TROG-D provides norm values for German-speaking children, these norms are based on a substantially smaller number of children than contained in this study and do not include children speaking Austrian varieties of German. Therefore, we used the sample percentiles to identify the bottom 10% (-1.25 SD) of the TROG-D scores. Based on three age groups (48–50 months, 51–56 months, and 57–62 months), percentiles were estimated using a continuous norming approach as implemented in the Cnormj package (41) in jamovi 1.6 (42).

(3) The AWST-R [Revised Active Vocabulary Test for 3- to 5-year-old children, Aktiver Wortschatztest für 3- bis 5-Jährige, Revision; (43)] is a standardized picture-naming test for the age range from 3;0 to 5;5 years. The items are ordered by increasing difficulty. To reduce the length of the assessment, we only used the first of the two picture folders (35 items) for the assessment of expressive vocabulary. As the AWST-R lacks norm values for the reduced version of 35 items, we again estimated norm values based on the study data. We once more applied a continuous norming approach. Screening scores in the bottom 10% were considered atypical.

Based on our definition, children with atypical scores (≤ −1.25 SD) in at least two of the reference tests were classified as LD. This applies to 38 children (10.2%).

#### Feasibility

A short questionnaire (7 items) was developed for screeners to assess time economy, acceptance of the screening materials by children and test administrators, practicability of LOGiK-S within the constraints of the universal screening procedure in the pre-school setting, ease of administration and estimation of sensitivity. Finally, testers were asked whether they would recommend the screening to others. All items were coded by use of three-point Likert scales, except the last one (yes-no answer). Due to the high similarity of the materials and procedures for children with German as their dominant language and children with a first language other than German no separate versions of the feasibility questionnaire were completed by the screeners. Only for information on screening time specific information relating exclusively to the LOGiK-S version for children speaking dominantly German was collected.

### Procedures

The screening procedures for both cohorts (A and B) were carried out by the speech and language pathologists, who usually conduct the annual universal language screening for children in their penultimate year in pre-school. The assessments were performed with each child individually in a separate room of their pre-school. The RG scale was introduced by a practice item to ensure the child's comprehension of the task and it was administered first, because it is usually perceived as less demanding or threating as no language production by the child is required. Within a maximum of 90 days, language development of the children was tested by use of standardized reference tests. The tests were administered in the pre-schools by experienced language experts from the Institute of Neurology of Senses and Language, who were blinded to the screening results.

### Statistical Analyses

First, we report descriptive statistics for the subscales. Second, we report reliability estimates (Kuder-Richardson KR-20) for the screening scales. Third, to evaluate construct validity of the screening scales, we applied confirmatory factor analysis (CFA) for binary items using a weighted least squares estimation (WLSMV) in Mplus 8 (44). Following the guidelines proposed by (41, 45) a good model fit is indicated by χ^2^/df ≤ 2, CFI ≥ 0.97, RMSEA ≤ 0.05. An acceptable fit is indicated by χ^2^/df ≤ 3, CFI ≥ 0.95, RMSEA ≤ 0.08. Fourth, to evaluate criterion validity, receiver operator characteristic (ROC) analyses were used to evaluate the diagnostic accuracy of the subscales. Following Swets (46), AUCs ≥ 0.9 are regarded as excellent, AUCs ≥ 0.8 and <0.9 as good, AUCs ≥ 0.7 and <0.8 as fair, and tests with AUCs <0.7 as poor. To compare AUCs of the subtests, we used a bootstrapped test for paired ROC curves—as implemented in the pROC package (47) in R. Fifth, we applied logistic regression using Jamovi 1.6 (42) to investigate whether both subscales independently contribute to the prediction of LD. Sixth, to evaluate the generalizability of the screening results we compared ROC curves between subsample (Cohort A vs. cohort B, boys vs. girls, age groups). As noted by Youngstrom (48) significant differences between subsamples would indicate variations in the diagnostic accuracy and thus, limit the generalizability of the screening results. A bootstrapped test for unpaired ROC curves was used to compare the AUCs between subgroups. Additionally, the Venkatraman permutation test (49) was used that compares actual ROC curves—not AUCs. If two ROC curves do not differ significantly, each cutoff values would result in the same sensitivity and specificity for the subsamples and therefore, a single cutoff would be appropriate for both subsamples. Finally, we used the R-OptimalCutpoints package (50) to determine appropriate cutoff scores. Cutoff scores are evaluated using the following diagnostic accuracy statistics: sensitivity (Se), specificity (Sp), positive predictive values (PPV), negative predictive values (NPV), and diagnostic likelihood ratios for positive and negative screening results (DLR+ and DLR–, respectively). Se and Sp ≥ 0.90 indicate good diagnostic accuracy, and Se and Sp ≥ 0.80 are regarded as fair. Values below.80 indicate an unacceptably high rate of misclassification (51). DLR+ indicates the multiplicative change in the pre-screening odds of having an LD given a positive screening result (i.e., post-screening odds = DLR+ × pre-screening odds) and DLR– is the change in the pre-screening odds of having an LD given a negative screening result (post-screening odds = DLR– × pre-screening odds). DLR+ values ≥ 10 and DLR– ≤ 0.1 indicate large changes in pre-screening odds, DLR+ ≤ 10 and > 5, and DLR– > 0.1 and ≤ 0.2 indicate moderate changes, DLR+ ≤ 5 and > 2, and DLR– > .2 and ≤ .5 indicate small changes. DLR+ <2 and DLR– > 0.5 are rarely important (52).

## Results

### Descriptive Statistics

**Figure 1:**
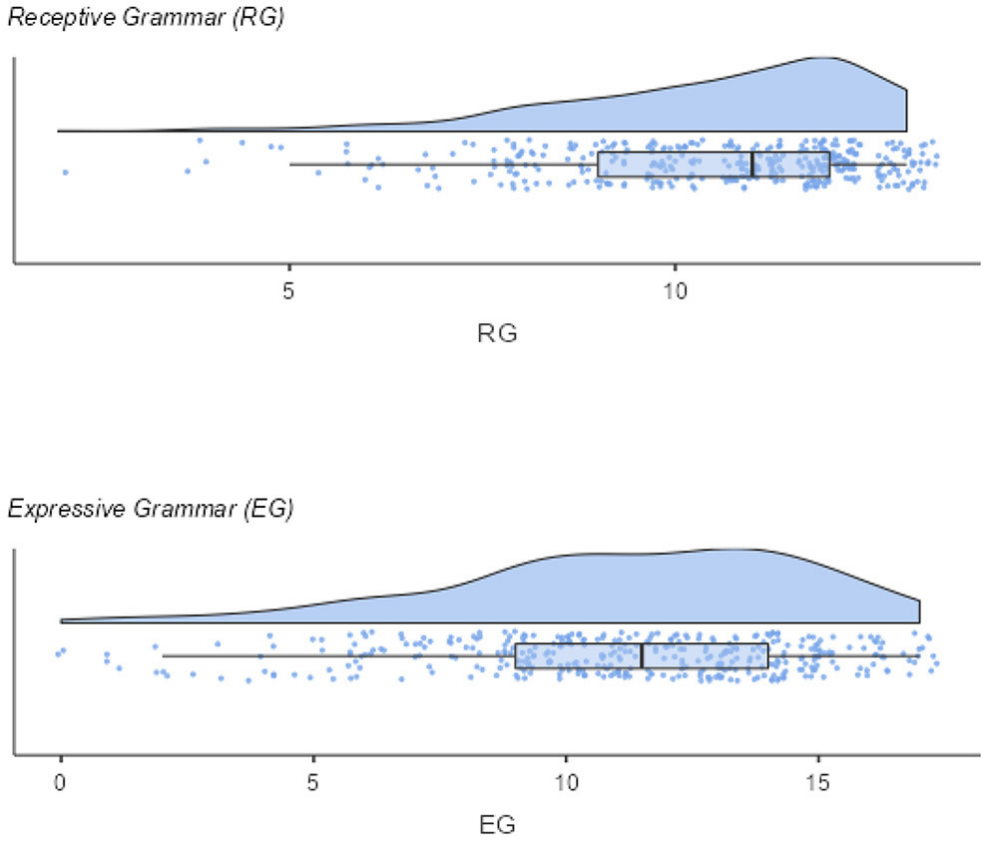
Distribution of the screening scales.

Figure 1 shows the distribution of the RG and EG screening subscales. As exactable for an LD screening, items are rather easy and thus, just a few children score in the bottom range of the screening scales. Consequently, the empirical means (MRG = 10.5, SDRG = 2.01; MEG = 11.2, SDEG = 3.57) are higher than the midpoints of the scales (RG = 6.5, EG = 8.5).

### Reliability

The internal consistency (KR-20) for the RG scale was rather low at .60. The internal consistency of the EG scale was of moderate size (KR-20 = .74).

### Construct Validity

We performed separate CFAs for the screening subscales. Overall, the CFAs for RG and EG yielded an acceptable fit (RG: χ^2^(65) = 90.046, *p* = 0.022, RMSEA = 0.032, CFI = 0.927, EG: χ^2^(119) = 245.049 (*p* < 0.001), RMSEA = 0.053, CFI = 0.898). However, for EG the CFI was quite low but also near the cutoff of 0.90 what is also sometimes considered as acceptable [e.g., (53)]. Next, we compared a two-factor model (EG and RG) with a one-factor model (i.e., all EG and RG items load on a single factor). The two-factor model yielded an acceptable to good fit (χ^2^(404) = 528.532, *p* < 0.001, RMSEA = 0.029, CFI = 0.921). The fit for the one-factor model was somewhat worse (χ^2^(405) = 568.600, RMSEA = 0.033, *p* < 0.001, CFI = 0.896). Notably, a χ^2^-difference tests indicated that the two-factor fits the data significantly better than the one-factor model [Δχ^2^(1) = 18.406, *p* < 0.001]. Overall, these results indicate that EG and RG are distinct but highly correlated constructs (latent correlation = 0.740, *p* < 0.001).

### Criterion Validity—Diagnostic Accuracy of Subscales

The EG subscale yielded an excellent AUC of .918 (Delong 95%-CI [0.881, 0.954]). The AUC for the RG subscale (AUC = 0.826; Delong 95%-CI [0.749, 0.902]) was good, but—as indicated by a bootstrapped test for AUC-differences—significantly smaller than the AUC for EG (D = −2.567, *p* < 0.05).

### Logistic Regression

A logistic regression showed that both subscales independently contribute to the prediction of LD (EG: b = −0.430, *p* < 0.001; OR = 0.650. RG: b = - 0.412, *p* < 0.001, OR = 0.662). McFadden's R^2^ was .433. Notably, as coefficients (bs and odds ratios) for EG and RG were quite equal, an increase of 1 in both subscales is associated with a similar increase in the risk for LD. Thus, a simple sum of RG and EG is an appropriate and easy to calculate (and thus, feasible) total screening score. The AUC for the total screening score was excellent (AUC = 0.928, DeLong 95%-CI = [0.888, 0.976]).

### Diagnostic Accuracy Differences Between Subgroups

The results of the comparisons of unpaired ROC curves (based on the total screening score) between subsamples are shown in Table 2. AUCs were generally excellent in all subsamples (only in the group of children younger than 56 months, the AUC was just below the limit of 0.90). Moreover, as indicated by insignificant group differences in AUCs (bootstrapped test for unpaired ROC curves) as well as in actual ROC curves (Venkatraman test), the diagnostic accuracy did not differ between the subsamples.

**Table 2:** Tests for unpaired ROC curves.

	**AUC**	**95%-CI (DeLong)**	**Comparision^**a**^**
**B—comparing cohorts**		
(1) Cohort A (2012)	0.945	[0.900, 0.991]	
(2) Cohort B (2013)	0.905	[0.841, 0.958]	E = 0.006, *p* > 0.05; D = 0.883, p > 0.05
**C—comparing age-groups**		
(1) younger than 56 months	0.899	[0.822, 0.977]	
(2) 56 months and older	0.967	[0.939, 0.996]	E = 0.013, *p* >0 .05; D = −1.618, p > 0.05
**D–comparing boys and girls**		
(1) boys	0.913	[0.856, 0.970]	
(2) girls	0.945	[0.893, 0.997]	E = 0.007, *p* >.05; D = – 0.833, *p* > 0.05

*^a^The first test statistic E refers to the Venkatraman test for unpaired ROC curves. The second test statistic D refers to a bootstrapped test for unpaired ROC curves*.

### Cut-Off Estimation

Finally, to determine an optimal cut-off, we used the “SpEqualSe” criterion (i.e., specificity equals sensitivity) in the Optimal Cutoff R-Package (50). At a cut-off of 18 (21, 8% screening fails) yielded a sensitivity of 0.842 (95%-CI = [0.687, 0.940]) and a specificity of 0.853 (95%-CI = [0.810, 0.889]). The PPV was 0.395 (95%-CI = [0.325, 0.656]) and the NPV was 0.979 (95%-CI = [0.951, 0.985]). DLR+ and DLR– were of moderate size. DLR+ was 5.722 (95%-CI = [4.270, 7.671]) and DLR– was 0.185 (95%-CI = [0.089, 0.386]).

### Feasibility

The 7-item questionnaire on feasibility of the LOGiK-S language screening, including both versions for children with German and Non-German as their dominant language and a phonology scale was returned by 39 (93%) from a total of 42 speech-language-therapists.

The average screening time was 9.49 min (SD 3.49). Screening materials were rated as very appealing by 44% and as appealing by 54%. Similarly, practicability within the constraints of universal language screening in the pre-school setting was rated as very good by 49% and as good by 46% of the respondents. Sensitivity (ie correct identification of children with LD) of LOGiK-S was assessed as very good by 15% and good by 80%. Thirty-nine percent described no personal effort in administering LOGiK-S, and another 90% stated low effort. As compared to the former screening without standardized measures 74% did not feel stressed at all by the new procedure whereas the rest reported minimal strain. Ninety-two percent would recommend the new measure to others.

## Discussion

This study investigated the performance (accuracy and feasibility) of the new screening measure LOGiK-S in a sample of two cohorts of 374 children in total, having German as their only or dominant language and attending the penultimate year of a public pre-school in Upper Austria. To avoid bias, the whole study sample that had been screened underwent testing by use of standardized language tests by speech-language experts blinded for the screening results. Screening results of the first cohort and practical experiences of the screeners were used to systematically reduce the number of screening items. Finally, all available data for the final selection of screening items were analyzed.

The EG scale of LOGiK-S demonstrated excellent accuracy (AUC = 0.918). The AUC of the RG scale was significantly smaller but still good (0.826). As indicated by logistic regression, both scales independently predict LD. A total screening score (combining EG and RG) showed excellent accuracy (AUC = 0.928). Using a cut-off of 18, the rate of screening fails was 21.8 %. Sensitivity (0.842) and specificity (0.853) were found to be good. As predictive values depend on the prevalence of the disorder under investigation (48), the rather low PPV (0.395) is not surprising given only 10.2 % of LD in our sample. Diagnostic likelihood ratios for positive and negative screening results (DLR+ and DLR–) of moderate size were found. Even though a higher PPV would be desirable as it leads to an overreferral of children, the dimensional nature of LDs must be taken into account. Children with false-positive screening scores have been shown to perform significantly lower on subsequent standardized measures than children with true-negative results (54) linked with a higher risk for language, psycho-social and cognitive delay. Therefore, follow-up diagnostic testing should be regarded as an opportunity to identify children with unmet needs for interventions (educational language and social support).

Tests for comparing unpaired ROC curves demonstrated no significant difference in screening accuracy (AUC and actual ROC curves) between both cohorts, despite some diversity in the study characteristics. Similarly, AUCs and ROC curves did not significantly differ between boys and girls and between younger and older children. Therefore, age related norms or sex related norms are not required. Overall, the independence of screening accuracy between groups (cohorts, sex, age) can be regarded as strengths of the screening instrument, as it supports its generalizability and therefore implementation with a variety of children and pre-schools can be recommended.

Feasibility of the new screening procedure was mostly rated as good or very good. Average screening time was below 10 min, materials were reported to be appealing to the children. Practicability within the constraints of the universal pre-school screenings was rated as very good and good. No or minimal personal effort involved in the administration of the new standardized instrument was described, and more than 90% of the screeners, who had to adapt their screening procedure to the new instrument, would recommend LOGiK-S to others.

Due to the lack of an accurately defined gold standard for LD in the literature we operationalized LD by language skills of at least 1.25 standard deviations below the norm in at least two of three linguistic dimensions following common practice in the field. Nevertheless, uncertainties of definition of the reference criterion must be considered a limitation. Moreover, a slight overrepresentation of children of parents with a university degree cannot be ruled out since population data for the specific target group (i.e., parents of children growing up monolingually in Upper Austria) are not available. Finally, the socioeconomic description of the sample is limited to parental education, because it was not possible to collect data on family income.

## Conclusion

The LOGiK-S is the first validated language screening measure that identifies increased risk of LD in children with Austrian German as their first or dominant language in their penultimate year of pre-school. Accuracy of LOGiKS was found to be high. ccuracy. Implementation with a variety of screeners and in a variety of pre-schools confirms high feasibility of the new measure. Consequently, the implementation of LOGiK-S for universal language screening can be recommended in Austria.

## Data Availability Statement

The dataset presented in this article is not readily available because parents have not given their consent to data sharing. Requests to access the dataset should be directed to daniel.holzinger@bblinz.at.

## Ethics Statement

The studies involving human participants were reviewed and approved by Ethikkommission Barmherzige Schwestern und Barmherzige Brüder. Written informed consent to participate in this study was provided by the participants' legal guardian/next of kin.

## Author Contributions

DH: conceptualization, funding acquisition, and supervision. DH and CW: methodology. BD: validation, investigation, and data curation. CW: formal analysis. DH, BD, and CW: writing—review and editing. DH and BD: project administration, and writing—original draft preparation. All authors have read and agreed to the published version of the manuscript.

## Funding

This work was supported by the Department of Social Affairs of the Upper Austrian Government. Article processing charge is funded by the Johannes Kepler University Open Access Publishing Fund.

## Conflict of Interest

The authors declare that the research was conducted in the absence of any commercial or financial relationships that could be construed as a potential conflict of interest.

## Publisher's Note

All claims expressed in this article are solely those of the authors and do not necessarily represent those of their affiliated organizations, or those of the publisher, the editors and the reviewers. Any product that may be evaluated in this article, or claim that may be made by its manufacturer, is not guaranteed or endorsed by the publisher.
